# Cold War Legacy in Public and Private Health Spending in Europe

**DOI:** 10.3389/fpubh.2018.00215

**Published:** 2018-08-06

**Authors:** Mihajlo Jakovljevic, Carl Camilleri, Nemanja Rancic, Simon Grima, Milena Jurisevic, Kenneth Grech, Sandra C. Buttigieg

**Affiliations:** ^1^Department of Global Health, Economics and Policy, Faculty of Medical Sciences, University of Kragujevac, Kragujevac, Serbia; ^2^Department of Economics, Faculty of Economics, Management and Accountancy, University of Malta, Msida, Malta; ^3^Centre for Clinical Pharmacology, Faculty of Medicine of the Military Medical Academy, University of Defence, Belgrade, Serbia; ^4^Department of Insurance, Faculty of Economics, Management and Accountancy, University of Malta, Msida, Malta; ^5^Department of Pharmacy, Faculty of Medical Sciences, University of Kragujevac, Kragujevac, Serbia; ^6^Department of Health Services Management, Faculty of Health Sciences, University of Malta, Msida, Malta; ^7^Clinical Performance Unit, Mater Dei Hospital, Msida, Malta

**Keywords:** public health, private health, health spending, cold war, Europe

## Abstract

Cold War Era (1946–1991) was marked by the presence of two distinctively different economic systems, namely the free-market (The Western ones) and central-planned (The Eastern ones) economies. The main goal of this study refers to the exploration of development pathways of Public and Private Health Expenditure in all of the countries of the European WHO Region. Based on the availability of fully comparable data from the National Health Accounts system, we adopted the 1995–2014 time horizon. All countries were divided into two groups: those defined in 1989 as free market economies and those defined as centrally-planned economies. We observed six major health expenditures: Total Health Expenditure (% of GDP), Total Health Expenditure (PPP unit), General government expenditure on health (PPP), Private expenditure on health (PPP), Social security funds (PPP) and Out-of-pocket expenditure (PPP). All of the numerical values used refer exclusively to per capita health spending. In a time-window from the middle of the 1990s towards recent years, total health expenditure was rising fast in both groups of countries. Expenditure on health % of GDP in both group of countries increased over time with the increase in the Free-market economies seen to be more rapid. The steeper level of total expenditure on health for the Free-market as of 1989 market economies, is due mainly to a steep increase in both the government and private expenditure on health relative to spending by centrally-planned economies as of the same date, with the out-of-pocket expenditure and the social security funds in the same market economies category following the same steepness. Variety of governments were leading Eastern European countries into their transitional health care reforms. We may confirm clear presence of obvious divergent upward trends in total governmental and private health expenditures between these two groups of countries over the past two decades. The degree of challenge to the fiscal sustainability of these health systems will have to be judged for each single nation, in line with its own local circumstances and perspectives.

## Introduction

Evolution of health care associated expenditure in Europe (1), like elsewhere, was closely related to the geopolitical and economic realities on the continent (2). Cold War Era lasting approximately from 1946–1991 was marked by the presence of two distinctively different economic systems, namely the free-market and central-planned economies. These two patterns of governance had also profoundly different views over the societal role of health care (3).

The Western ones, led by the USA, were so called free-market economies and their dominant social theory ultimately leading to the rise of neoliberal capitalism. In health care, a variety of models were deployed but Beveridge and Bismarck models of health care financing and provision were the most broadly accepted (4). It is very important to emphasize that the return on investment in health care and the role of population health in societal economic productivity were well understood very early on by the prominent Western health economists (5). This knowledge was later on successfully introduced into the social policy. The level of medical technology and innovation, with few exceptions among some disciplines, tended to be higher compared to the East. However major weaknesses of these systems were rather significant, with social inequities in terms of access to medical care and affordability. These inequities, in some leading Western health systems (6), became even deeper with the accelerated globalization (7) that followed after the end of the Cold War.

The Eastern ones, led by the USSR, were presented by centrally-planned socialist economies that were rooted in Marxist social theory. The Soviet Semashko model of health care financing and provision prevailed in these countries. To its great historical credit, it is recognized to be the first one to globally deliver universal health coverage back in the early 1930s at the level of medical technology of that time. Even the poorest citizens had the right to state-funded basic medical care (8). After WWII, the famous Five-year plans led to rapid industrialization in USSR and some of its client states. This ultimately established USSR as the second ranked economy globally (9) for the most of Cold War Era duration (10). It is important to notice that both health care and education were regarded as purely consumption branches of the overall economy (11). They were assigned limited resources unlike some industrial priority areas believed to be far more productive in bringing budgetary revenues (12). This causal link between population health and social economic productivity was not well understood, and in reality not even exploited. Medical technology development and pace of innovation, with limited exceptions in some cutting-edge disciplines [psychiatry (13), orthopedic (14) and eye surgery (15), cosmic (16), aeronautic and alternative medicine (17) to mention a few (18)] were lagging behind vis-à-vis the West (19). However the social justice system in the East was exceptionally efficient (20). Poverty was almost eradicated and social inequalities in terms of access to state-funded health care were far lower compared to the Western ones (21). The scale of corruption and informal payments within the health system at that time were controlled and rather low (22). These countries became heavily industrialized, characterized by massive rural-urban migration and morbidity and mortality structures were similar to the West (23). Although the pool of maternal mortality was liquidated (24) and early childhood survival (25) improved rapidly in early post-WWII decades (26), overall life expectancy was lagging significantly behind the top performing free market economies (27).

## Methods

The main goal of this study refers to the exploration of development pathways of Public and Private Health Expenditure in all of the countries of the European WHO Region following their different starting points back in time at the end of the Cold War Era (28). Back in 1991 free-market economies continued evolving their traditions further and accelerated globalization was one of the main changes affecting health policy challenges. Unlike them, since 1991 Central and Eastern European centrally planned socialist economies underwent profound and complex socioeconomic and health care reforms. Their aim was to convert old socialist into a new capitalism grounded economic system (29). At the same time, mostly less efficient, massive, hospital, curative-oriented health systems had to be changed into the lighter and less costly ones based on preventive medicine (30) and outpatient care (31). These processes of social change became broadly known as the “Eastern European Transition” (32). In some countries of the region, they came almost to an end in 2017, while in others they continued with less or more significant changes of health policy and financing traditions. It should be noted that some countries of this region among the Commonwealth of Independent Nations (CIS) led by Russian Federation, after the early attempts in 1990s (33), have willingly abandoned such transition and adopted their own distinctive model of development, based on Semashko traditions (34).

Based on the availability of fully comparable data from the National Health Accounts system (35) introduced by WHO, we adopted the 1995–2014 time horizon. After thorough consideration of several public registries issued by the UN, OECD, World Bank, EuroStat and other multilateral agencies, we decided that the Global Health Expenditure Database will be our sole source of data for this study (36). We took the end of the Cold War as a point in time when initially divergent economic models began to converge in certain number of countries. What we wanted to show is that even today, after two and a half decades of “transition,” countries eastern from the Iron Curtain still in many core indicators of health spending are closer to their Semashko root than to the Western Bismarck/Beveridge model like in the pharmaceutical spending for example the studies of Álvarez-Gálvez,and Jaime-Castillo in 2018 (37). Although, divergency began in 1917 after the Revolution, during the Westfallen peace in between two world wars most of Central Europe was still capitalist.

The initial set of observed variables comprised of ten different health spending indicators: Public funds, Rest of the world funds / External resources, Total expenditure on health, General government expenditure on health, Ministry of Health, Social security funds, Private expenditure on health, Private insurance, Out-of-pocket expenditure and Non-profit institutions serving households (e.g., NGOs). However, after a pilot extraction of data was done, we noticed significant gaps in both chronology and geographical coverage. These could not be addressed with any valid statistical missing data handling strategy. Therefore, we shortlisted the final count to the six major health expenditures, all of which were broadly presented and available: Total Health Expenditure (% of GDP), Total Health Expenditure (PPP unit), General government expenditure on health (PPP), Private expenditure on health (PPP), Social security funds (PPP), and Out-of-pocket expenditure (PPP). All of the numerical values used refer exclusively to per capita health spending in order to eliminate the bias arising from any nation's population size.

Using the premise of this observation, all countries were divided into two groups: those defined in 1989 as free market economies and those defined as centrally-planned economies. With the exception of Eastern Germany after reunification, data on all other UN recognized countries were accessible regardless of the changes of borders and statehoods in Central and Eastern Europe (38). A clear list of countries in both groups can be found in Table 1 below. Moreover, Table 2 below, shows the data for each individual country within both groups.

**Table 1:** Division of European countries based on their economic system at the end of Cold War Era back in 1989.

**Free market economies as of 1989**	**Centrally planned economies as of 1989**
Andorra Austria Belgium Cyprus Denmark Finland France Germany Greece Iceland Ireland Israel Italy Luxembourg Malta Monaco Netherlands Norway Portugal San Marino Spain Sweden Switzerland Turkey United Kingdom of Great Britain and Northern Ireland	Albania Armenia Azerbaijan Belarus Bosnia and Herzegovina Bulgaria Croatia Czech Republic Estonia Georgia Hungary Kazakhstan Kyrgyzstan Latvia Lithuania Montenegro Poland Republic of Moldova Romania Russian Federation Serbia Slovakia Slovenia Tajikistan The former Yugoslav Republic of Macedonia Turkmenistan Ukraine Uzbekistan

**Table 2:** The data for each individual country within both groups.

**Median (95% confidence intervals)**	**Total expenditure on health (% of GDP)**	**Total expenditure on health in current PPP per capita**	**General government expenditure on health in current PPP per capita**	**Private expenditure on health in current PPP per capita**	**Out of pocket expenditure in current PPP per capita**	**Social security funds in current PPP per capita**
**FREE MARKET ECONOMIES AS OF 1989 (1995–2014)**						
Andorra	6.1 (6.0–7.3)	2259.7 (2146.2–3028.5)	1587.4 (1507.4–2293.1)	717.9 (627.0–747.2)	519.7 (444.9–538.3)	1222.6 (1170.5–1712.5)
Austria	10.4 (10.2–10.7)	3517.7 (3203.0–4011.7)	2605.8 (2394.2–3019.4)	912.0 (807.4–993.7)	613.9 (514.8–658.5)	1516.6 (1382.2–1712.2)
Belgium	9.2 (8.7–9.6)	2949.7 (2601.4–3406.7)	2262.7 (1977.4–2616.1)	706.8 (622.6–792.0)	559.1 (503.6–633.7)	1931.4 (1686.7–2238.2)
Cyprus	6.3 (6.0–6.8)	1555.4 (1340.1–1828.9)	671.8 (567.9–811.9)	889.8 (769.5–1015.4)	727.5 (691.1–883.0)	–
Denmark	9.7 (9.2–10.2)	3188.3 (2899.9–3815.4)	2690.1 (2438.0–3235.2)	498.2 (461.7–580.5)	450.6 (417.0–515.9)	–
Finland	8.2 (8.0–8.6)	2525.2 (2220.0–2911.2)	1857.5 (1626.0–2167.9)	667.6 (593.8–743.6)	512.1 (457.5–561.7)	373.2 (312.6–413.9)
France	10.5 (10.3–10.8)	3159.9 (2885.6–3597.1)	2462.9 (2266.6–2799.4)	697.0 (615.3–785.5)	221.9 (205.7–251.7)	2357.3 (2149.4–2661.6)
Germany	10.4 (10.2–10.7)	3283.7 (3076.4–3891.5)	2504.8 (2404.6–2993.1)	780.1 (670.5–899.6)	458.0 (381.4–517.2)	2190.9 (2095.2–2640.2)
Greece	8.7 (8.5–9.1)	2096.9 (1837.3–2343.5)	1266.4 (1090.0–1475.4)	810.5 (734.2–878.5)	710.1 (648.0–784.5)	642.5 (494.4–791.9)
Iceland	8.9 (8.7–9.2)	3338.7 (2842.2–3357.9)	2728.0 (2323.4–2735.0)	600.0 (517.5–624.2)	550.8 (486.5–577.8)	910.9 (780.2–937.6)
Ireland	7.2 (6.9–7.8)	2901.8 (2306.3–3204.3)	2208.6 (1685.3–2303.6)	693.1 (609.6–912.1)	451.0 (362.6–525.1)	14.1 (11.4–16.7)
Israel	7.4 (7.4–7.5)	1871.6 (1785.1–2074.6)	1174.6 (1131.1–1292.9)	678.9 (620.2–764.8)	512.7 (472.9–549.9)	826.4 (818.5–939.3)
Italy	8.5 (8.1–8.8)	2520.5 (2290.7–2836.1)	1913.7 (1690.4–2140.3)	608.9 (598.7–697.4)	538.5 (533.4–607.1)	2.3 (2.4–5.3)
Luxembourg	7.3 (6.7–7.5)	5420.5 (4171.4–5656.7)	4600.2 (3618.7–4831.3)	778.2 (544.5–830.2)	573.3 (415.6–599.5)	3662.8 (2938.5–3936.9)
Malta	8.2 (7.4–8.6)	1906.0 (1574.7–2206.7)	1284.2 (1057.5–1471.8)	621.8 (514.9–737.3)	536.6 (461.9–663.2)	–
Monaco	3.7 (3.5–3.9)	4269.9 (3625.2–4836.9)	3762.8 (3194.6–4266.0)	507.1 (429.8–571.7)	298.9 (253.8–338.6)	3707.6 (3140.8–4204.8)
Netherlands	8.9 (8.4–9.6)	3302.9 (2908.8–3996.6)	2236.5 (2131.6–3269.4)	673.1 (673.4–831.0)	237.0 (210.7–247.0)	2069.1 (1966.3–2981.0)
Norway	9.1 (8.6–9.2)	4204.1 (3556.6–4835.9)	3512.6 (2976.2–4080.2)	691.6 (579.4–755.9)	658.6 (551.7–716.1)	–
Portugal	9.5 (8.8–9.6)	2101.7 (1752.4–2301.8)	1472.6 (1179.9–1550.9)	629.1 (569.7–753.6)	459.2 (408.6–558.8)	–
San Marino	4.7 (4.5–5.2)	2700.7 (2626.2–3017.3)	2468.7 (2382.9–2780.1)	234.8 (227.3–253.1)	213.2 (206.4–229.8)	2468.7 (2382.9–2780.1)
Spain	8.1 (7.8–8.6)	2162.7 (1884.8–2506.1)	1562.0 (1363.9–1827.6)	600.8 (519.1–680.3)	463.8 (418.1–542.1)	134.4 (120.2–143.7)
Sweden	9.1 (8.8–10.0)	2964.9 (2696.4–3702.8)	2409.5 (2241.6–3079.7)	555.5 (450.3–627.5)	481.2 (404.2–550.7)	–
Switzerland	10.5 (10.3–10.9)	3988.9 (3723.2–4839.1)	2350.7 (2184.8–3026.8)	1638.2 (1530.6–1797.0)	1233.7 (1130.5–1361.8)	–
Turkey	5.3 (4.5–5.4)	587.1 (505.3–757.6)	407.6 (361.1–568.0)	169.2 (139.7–194.1)	125.6 (107.0–141.4)	244.8 (206.4–359.6)
United Kingdom of Great Britain and Northern Ireland	8.1 (7.6–8.6)	2653.1 (2189.4–2860.8)	2150.1 (1780.8–2348.2)	504.6 (403.6–514.8)	259.8 (226.8-279.2)	–
**CENTRALLY PLANNED ECONOMIES AS OF 1989 (1995**–**2014)**						
Albania	6.1 (6.0–6.5)	371.1 (329.8–448.8)	160.2 (128.8–204.0)	211.0 (200.6–245.3)	197.8 (196.2–240.0)	45.9 (49.1–123.1)
Armenia	5.3 (4.8–5.5)	233.8 (179.0–255.1)	73.0 (61.3–104.6)	140.8 (115.7–152.5)	134.1 (110.3–145.0)	–
Azerbaijan	5.4 (5.2–6.1)	492.0 (347.0–628.5)	59.0 (63.0–125.7)	429.8 (282.6–504.1)	384.4 (246.9–450.0)	–
Belarus	6.2 (5.9–6.4)	614.3 (491.2–726.8)	453.1 (355.5–511.2)	161.2 (132.7–218.6)	114.7 (96.0–178.3)	–
Bosnia and Herzegovina	8.7 (8.3–9.1)	536.3 (441.6–683.7)	306.9 (267.0–458.3)	229.3 (172.1–227.9)	229.3 (170.8–224.4)	290.8 (253.9–429.7)
Bulgaria	6.8 (6.1–7.0)	686.8 (561.6–881.3)	417.6 (339.7–500.7)	268.9 (221.4–381.1)	261.3 (216.9–371.1)	–
Croatia	7.2 (6.9–7.5)	1047.0 (949.7–1324.8)	876.1 (801.2–1110.3)	166.0 (145.8–217.2)	157.2 (131.1–175.7)	790.3 (724.6–967.8)
Czech Republic	6.8 (6.7–7.1)	1434.2 (1262.5–1672.8)	1265.0 (1109.9–1430.8)	169.2 (151.7–242.9)	151.0 (142.9–223.9)	1134.3 (994.7–1298.2)
Estonia	5.8 (5.5–6.0)	786.8 (725.3–1121.9)	599.1 (573.9–879.9)	184.7 (144.1–234.3)	164.0 (132.6–221.5)	–
Georgia	8.3 (7.5–8.6)	328.6 (277.0–448.9)	57.1 (48.0–85.0)	271.5 (228.1–364.8)	253.3 (202.2–301.5)	30.1 (25.7–54.5)
Hungary	7.5 (7.3–7.7)	1381.0 (1087.6–1455.6)	955.5 (769.6–980.1)	416.7 (316.8–476.8)	346.7 (266.0–379.4)	787.8 (639.0–817.2)
Kazakhstan	4.1 (3.9–4.3)	512.0 (435.9–670.7)	291.7 (254.1–382.3)	206.3 (179.9–290.3)	203.4 (177.4–286.7)	–
Kyrgyzstan	5.9 (5.7–6.3)	120.0 (109.8–155.5)	49.0 (52.0–82.7)	70.3 (57.2–73.4)	63.3 (52.1–66.4)	–
Latvia	6.2 (6.1–6.4)	569.2 (475.7–701.0)	323.5 (278.1–424.9)	245.7 (193.7–270.5)	231.6 (185.2–256.8)	–
Lithuania	6.3 (6.1–6.5)	816.7 (749.2–1165.1)	587.1 (533.7–811.2)	257.6 (210.2–341.2)	253.7 (202.2–332.0)	468.1 (400.3–664.4)
Montenegro	7.4 (7.0–7.7)	684.0 (601.1–778.6)	485.9 (407.1–504.0)	188.6 (189.7–278.9)	188.6 (189.7–278.9)	455.0 (390.6–473.5)
Poland	6.2 (6.0–6.4)	831.8 (770.9–1134.8)	573.5 (543.5–800.9)	257.6 (226.3–332.0)	225.3 (201.5–273.2)	–
Republic of Moldova	9.8 (8.7–10.3)	247.7 (225.5–355.9)	117.1 (113.4–173.9)	130.5 (111.3–182.8)	105.7 (91.1–150.5)	–
Romania	5.3 (4.5–5.2)	502.4 (421.7–716.1)	390.0 (336.4–574.5)	108.1 (84.5–141.6)	105.2 (83.0–138.2)	–
Russian Federation	5.9 (5.8–6.5)	573.3 (616.8–1101.4)	349.0 (371.1–616.8)	224.3 (244.8–485.5)	184.7 (203.4–444.8)	142.4 (144.0–266.0)
Serbia	8.5 (7.7–9.1)	719.6 (586.1–949.1)	484.4 (378.6–594.5)	235.1 (206.2–353.0)	206.7 (185.1–328.2)	446.2 (350.9–553.6)
Slovakia	7.1 (6.4–7.5)	1101.0 (966.7–1543.1)	815.8 (754.9–1107.9)	285.2 (209.2–437.8)	230.6 (172.8–341.9)	709.8 (696.2–1004.3)
Slovenia	8.5 (8.2–8.8)	1942.4 (1662.8–2177.5)	1423.9 (1228.8–1593.4)	520.7 (433.4–584.8)	233.5 (196.0–260.4)	1303.8 (1128.5–1447.9)
Tajikistan	5.2 (4.5–5.6)	81.0 (62.2–106.7)	15.4 (15.6–28.6)	65.6 (46.3–78.4)	63.6 (43.7–71.2)	–
The former Yugoslav Republic of Macedonia	8.1 (7.4–8.4)	638.5 (578.2–694.5)	378.2 (353.9–442.3)	240.6 (220.9–255.5)	240.6 (220.9–255.5)	365.1 (337.8–412.3)
Turkmenistan	3.1 (2.6–3.5)	172.3 (164.0–210.7)	118.1 (105.6–137.4)	62.1 (55.7–75.9)	62.1 (55.7–75.9)	–
Ukraine	6.7 (6.5–7.0)	410.2 (342.2–486.1)	242.1 (197.5–275.2)	168.1 (143.1–212.5)	156.0 (132.1–198.0)	–
Uzbekistan	5.7 (5.6–6.1)	133.4 (142.7–215.7)	61.5 (66.6–104.0)	74.1 (75.4–112.4)	69.7 (73.0–106.6)	–

*Sources: World Health Organization-Global Health Estimates-Database (WHO GHE DB)*.

## Statistical analysis

We then conducted a comparative statistical analysis on two time cross sections, comparing these two groups of countries for the period 1995–2014. Another part of the analysis refers to comparison of the time trend between the groups. For the first case, the Mann-Whitney U Test was applied and in another case we decided for the Wilcoxson's test, because our data did not fulfill parametric conditions for a normal (Gauss') distribution. We checked this fact with the Shapiro-Wilk test.

## Study limitations

This study presents a retrospectively designed research on aggregate national level data. Such data are being reported by the national authorities, such as governments and ministries of health to the respective UN and WHO offices. Authors take data as guaranteed by the national governments and checked by WHO European Office and are not capable of checking reliability, consistency of such reporting or the internal accounting systems which may slightly vary from country to country. This way of tracking and reporting financial flows within the nation's health system have been made as much consistent as possible through the lengthy process of WHO initiated introduction of the National Health Accounts in the early 1990s (39). It assumed mandatory staff trainings and capacity building by the health insurance funds' and ministry of health officials exactly for the purpose to make these follow the unique patterns and indicator definitions that have been adopted during the establishment of the NHA system by all the representatives of all the country members of the United Nations.

Other possible limitations refer to the fact that this is a purely health economic observation. While conducting this study, we were focused on the different dimensions of health expenditures of given European nations while using only six core indicators and only two units of measurement (THE as % of GDP and PPP) out of many currency units available in a given database (40). If we had opted to observe country group parities in nominal dollar terms, landscape might have looked quite different (41). However, we followed the ground health economics theory that says that purchasing power parity allows the best possible comparison among the nations with significantly different levels of income/industrial development (42). Likewise, observation of total health expenditure expressed as percentage point share of gross domestic product was selected, because according to broadly accepted economic theory, this indicator is the only one allowing us transnational comparisons among inherently different economic systems (43).

Based on data we worked with, there is no evidence for definite conclusions on effectiveness and performance of these national health systems in terms of their public health output. We did not use, nor consider data such as longevity, morbidity, mortality, utilization of medical services or medicines or any other similar indicators (44), as these were beyond the scope of this paper. Therefore, conclusions of this study are limited to health spending dynamics and its evolution over the long period of time without any referral to the success rates of individual systems or their cost-effectiveness / resource allocation efficiency (45).

## Results

This study has revealed a set of findings, which were not previously observed to a deeper extent in published evidence (46). In a time-window from the middle of the 1990s toward recent years, total health expenditure was rising fast in both groups of countries. While it almost quadrupled among former socialist countries, in a group of EU15 and few other similar nations, this growth was even more concerning. It began from four times higher starting point around $1,600 PPP on average within the group and reached a value of almost $4,200 PPP in only two decades.

The graphs below illustrate linear regression models of Total Health Expenditure % of GDP as a function of time (years). In general, the models for both the centrally-planned economies and the market-based economies fit the data well. The regression line for free-market economies has a steeper gradient than the one for centrally-planned economies. This is suggestive of accelerated rising costs over time in the former.

**Figure 1:**
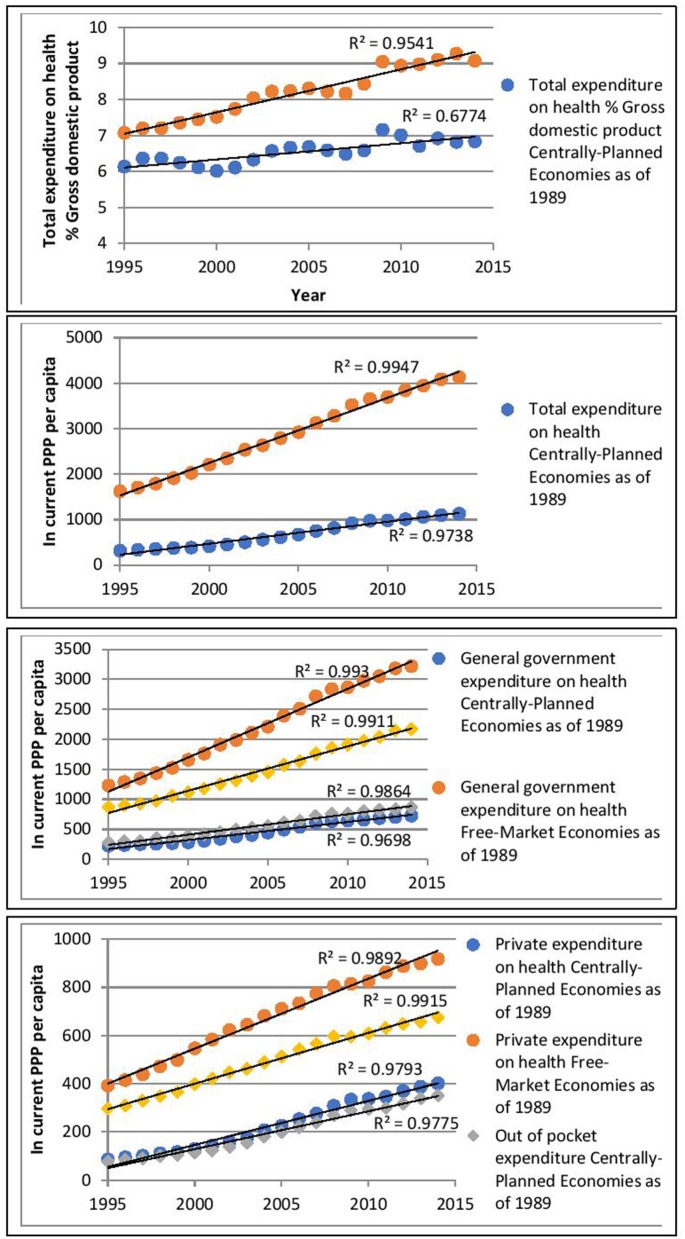
Long term upward trends of health expenditure data extrapolated on the entire group of countries as pondered average of annual values.

Figure 1 shows that total expenditure on health % of GDP in both group of countries increased over time with the increase in the Free-market economies seen to be more rapid. In fact, we can observe some form of similarity in the patterns of both lines. Moreover, the “wave” pattern in both lines seem to be identical for particular years. The level of total expenditure on health in free market economies, starts at a higher level, compared to the centrally planned countries and increases at a faster rate over the time period studied. This is suggestive of both types of economies being subjected to the same types of economic pressures and possibly to the strength of the prevailing global economy. Despite this, the free-market economies' spending remains steeper than the centrally-planned ones.

The steeper level of total expenditure on health for the Free-market as of 1989 market economies, is due mainly to a steep increase in both the government and private expenditure on health relative to spending by centrally-planned economies as of the same date, with the out-of-pocket expenditure and the social security funds in the same market economies category following the same steepness.

Moreover, a widening of the gap in expenditure between the two types of economies over time can be noted. Which seems to result from a relatively stable low level of social security funds in the centrally planned group over the years.

**Figure 2:**
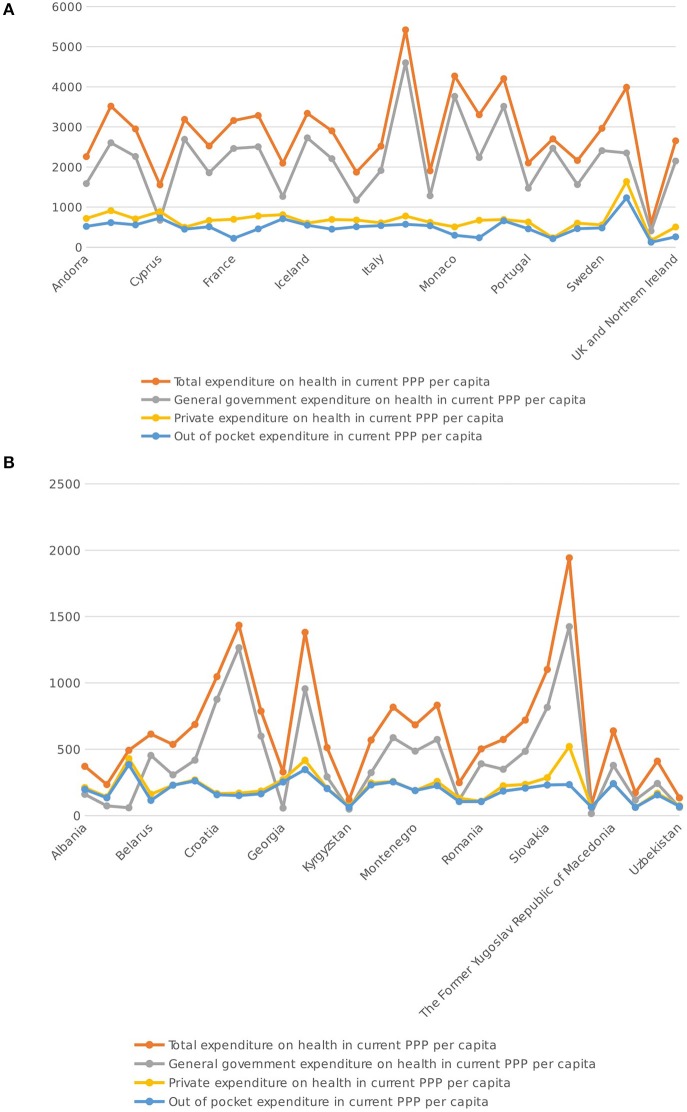
**(A)** Free Market Economy Indicator comparisons Source: World Health Organization- Global Health Estimates - Database (WHO GHE DB). **(B)** Centrally Planned Market Economy Indicator comparisons Source: World Health Organization- Global Health Estimates - Database (WHO GHE DB).

Although, the interest was to study the aggregate, results have also been evaluated and studied at an individual country level. When one compares the averages over the periods within the two figures in Figure 2 it is still clear that the levels within the “Free Market” economies is overall higher in comparison to the “Centrally Planned” economies. Moreover, when observing the outliers, Luxembourg, Monaco, Norway and Switzerland in one group of countries and Czech Republic, Hungary, and Slovenia in the other group, the range of variation across countries is also much larger in the “Free Market” economic group which seems to indicate much more variation within this group of countries over the years under study.

One can also note, from Figure 2, that there is little fluctuation in the private expenditure and the out-of-pocket variables being considered. This implies that variation arises mainly from differences in general government expenditure.

## Discussion

Since 1960s, it became apparent first in the US health system that average costs of medical care are rising faster than average monthly income of ordinary citizen. At the macroeconomic level, over time it became visible that this growth was almost twice faster compared to economic growth or gross domestic product disposable within a nation (47). Vast body of literature has identified as some of the major drivers of such growth: blossoming of non-communicable prosperity diseases (48), population aging (49), innovation in medicine (50) and pharmaceuticals in particular (51), excessive utilization of hospital diagnostic imaging (52), underutilization of primary care (53), and preventive measures and inefficient management (54) among others. This issue of financial sustainability of national health care systems became prominent in Western literature (55). Accordingly, to meet these challenges health economics as an interdisciplinary science emerged from American traditions in academic economics (56, 57).

On the other hand, during the Cold War Era, socialist countries controlled these health care costs at an unrealistic level by several ways (58). One of them was a negotiation process between one central state-owned health insurance fund as a major purchasing authority for health services and a large tertiary care hospitals as a core provider of such services. They used to be paid based on the performance such as total duration of hospital admissions, number of surgical procedures performed or outpatient physician examinations (59). However, due to the fact that these funds tended to generate debt in most countries and ongoing fiscal deficits, these services were not covered in total value, but just as a dominant share of such costs. For the rest, hospitals themselves had to generate revenues by a variety of ways but mostly by charging the difference as patient/citizen participation fees (60).

All of these weaknesses became more prominent after the beginning of socioeconomic transition (61) in Eastern Europe since 1991 (62). As these countries moved from the state controlled model toward market controlled mechanisms, a large degree of vulnerability occurred both for the citizens in need (63) and the health system itself (32). This all worsened to a large extent due to the Russian Federation's economic recession reaching its worst-ever level in 1998. This phenomenon dragged the entire region and central Asia into an ensuing economic crisis. This was followed by a notorious mortality crisis in Russia (64) and neighboring nations. Eventually, the situation rapidly improved in the early 2000s (65).

Difficulties experienced by the variety of national health systems in this region are closely explained in the published literature (66). Some authors even went as far as declaring some countries to be “winners” and others “losers” of transition (66). This in our opinion is exaggerated because, almost 30 years after, health policy observations, taught us that each single nation succeeded to adapt in its own way (67). Health coverage (68), accessibility and affordability of services and ultimately core population health outcomes such as longevity, all improved visibly in Eastern Europe (44).

It should be noted that countries created from former Yugoslav Republics present a rather distinct case (68). The former, Yugoslavia, geopolitically outside the Iron Curtain, was by far the richest socialist country. It deployed the system of health care provision and financing, which presented a mixture of Soviet Semashko and Bismarck traditions (69). Its community health outcomes were mostly outperforming other similar nations. After the civil wars of its dissolution in 1990s ended, most countries of the region entered this transition and health care reforms with approximately one decade delay (70). Their public health indictors today slightly lag behind Poland (71), Hungary (72) or Czech Republic (73). However, keeping in mind contemporary health spending disparity in favor of eastern EU members as of 2004, their health systems perform quite satisfactory (74).

Over the years the differences in both the levels of total expenditure on health (in PPP per capita terms) and the proportion of total health expenditure as a % of GDP across the two sets of market economies has increased. Both sets of market economies have recorded significant increases over the years within both components of interest. However the increasing variation between the two sets of countries is clearly noticeable. Indeed, at a more disaggregated level, both general government expenditure on health and private expenditure on health within free market economies reflect the significant increases recorded over the years. The developments within the expenditure on the social security funds component over the years also reflect the above considerations. Whilst recognizing that there might be divergences in behavior over time for such components, within the specific countries which make up each of the two groups under study, the general observations mentioned above apply for most of the particular countries in question.

## Conclusion

Variety of governments were leading Eastern European countries into their transitional health care reforms. This process was followed by difficult years of poverty, rising socioeconomic inequalities (75) and system inefficiencies to provide equitable and affordable medical care to the citizens (76). The ground assumption of the authorities at some point in time was that former socialist countries should converge with their Western counterparts both in terms of health spending and outcomes. We may witness that these goals have been met only to some extent (77). Long term trends even depict clear divergent trends in some health expenditure indicators. Similar phenomenon has already been described in pharmaceutical spending in previous findings (78). Judgment of allocative or technical efficiency of such financial policies is beyond this research. Although, we may say that historical free-market societies appear to be rising their ability to invest faster in health care (79), based on the data observed, we are unable to estimate the degree of success in public health indicators in particular nations. However, we may confirm clear presence of obvious divergent upward trends in total governmental and private health expenditures between these two groups of countries over the past two decades. The degree of challenge to the fiscal sustainability of these health systems will have to be judged for each single nation, in line with its own local circumstances and perspectives (80).

## Author contributions

MJ and SB jointly designed the study and defined research questions. NR did most of the data mining and extraction, purification of files for missing data and artifacts and statistical analysis. CC and SG contributed to the tables, figures creation, and interpretation of data. SG structured, coordinated and uploaded the final revised paper. MJ and KG contributed in the initial discussion. MJ drafted the working version manuscript but all authors contributed to the final version to the extent of important intellectual content.

### Conflict of interest statement

The authors declare that the research was conducted in the absence of any commercial or financial relationships that could be construed as a potential conflict of interest.

## References

[1] Mackenbach JP. Political conditions and life expectancy in Europe, 1900–2008. Soc Sci Med. (2013) 82:134–46. 10.1016/j.socscimed.2012.12.022 23337831

[2] Getzen TE. Medical care price indexes: theory, construction and empirical analysis of the US series 1927–1990. Adv Health Econ Health Serv Res. (1992) 13:83–128. 10129447

[3] Global Burden of Disease Health Financing Collaborator Network Evolution and patterns of global health financing 1995–2014: development assistance for health and government, prepaid private, and out-of-pocket healthspending in 184 countries. Lancet (2017) 389:1981–2004. 10.1016/S0140-6736(17)30874-7 28433256 PMC5440770

[4] Wallace LS. A view of health care around the world. Ann Fam Med. (2013) 11:84. 10.1370/afm.1484 23319511 PMC3596027

[5] Musgrove P. Investing in health: the 1993 world development report of the world bank. Bull Pan Am Health Organ. (1993) 27:284–6. 8220523

[6] Griffin K Khan AR Globalization and the Developing World: An Essay on the International Dimensions of Development in the Post-Cold War Era (July 16, 1992). UNDP Human Development Report Office Paper No. 2. (1992). Available online at https://ssrn.com/abstract=2294619

[7] Labonte R Schrecker T Packer C Runnels V Globalization and Health: Pathways, Evidence and Policy. Howick Place, London: Routledge (2009).

[8] Semashko NA Health Protection in the USSR. London: Victor Gollancz (1934).

[9] Maddison A Contours of the World Economy 1-2030 AD: Essays in Macro-Economic History. Newyork, NY: Oxford University Press (2007).

[10] Wikipedia List of Countries by Largest Historical GDP (2016). Available online at https://en.wikipedia.org/wiki/List_of_countries_by_largest_historical_GDP

[11] Kesternich I Siflinger B Smith JP Winter JK. The effects of world war ii on economic and health outcomes across Europe. Rev Econ Stat. (2014) 96:103–18. 10.1162/REST_a_00353 24850973 PMC4025972

[12] Lambelet D The contradiction between Soviet and American human rights doctrine: reconciliation through perestroika and pragmatism. BU Int L J. (1989) 7:61 https://scholarship.law.duke.edu/cgi/viewcontent.cgi?referer=https://www.google.rs/&httpsredir=1&article=1744&context=faculty_scholarship

[13] Zajicek B Scientific Psychiatry in Stalin's Soviet Union: The Politics of Modern Medicine and the Struggle to Define ‘Pavlovian’ Psychiatry, 1939–1953, Dissertation, The University of Chicago (2009). 507:3369465.

[14] Green SA. Ilizarov orthopedic methods: innovations from a Siberian surgeon. AORN J. (1989) 49:215–23. 10.1016/S0001-2092(07)67485-7 2913937

[15] Ghaffariyeh A Honarpisheh N Karkhaneh A Abudi R Moroz ZI Peyman A. Fyodorov–Zuev keratoprosthesis implantation: long-term results in patients with multiple failed corneal grafts. Graefe's Arch Clin Exp Ophthalmol. (2011) 249:93–101. 10.1007/s00417-010-1493-8 20798954

[16] Doarn CR Nicogossian AE Grigoriev AI Tverskya GJ Orlvo OI Ilyin EA A summary of activities of the US/Soviet-Russian joint working group on space biology and medicine. Acta Astronautica (2010) 67:649–58. 10.1016/j.actaastro.2010.05.011

[17] Pchelnikov YN Kholodnyi VA Medical application of surface electromagnetic waves. Bioelectrochem Bioenerg. (1998) 47:283–90. 10.1016/S0302-4598(98)00200-1

[18] Konstantinov IE. At the cutting edge of the impossible: a tribute to Vladimir P. Demikhov. Tex Heart Inst J. (2009) 36:453–8. 19876428 PMC2763473

[19] Field MG. Noble purpose, grand design, flawed execution, mixed results: Soviet socialized medicine after seventy years. Am J Public Health (1990) 80:144–5. 10.2105/AJPH.80.2.144 2404421 PMC1404602

[20] Krieger N Birn AE. A vision of social justice as the foundation of public health: commemorating 150 years of the spirit of 1848. Am J Public Health (1998) 88:1603–6. 10.2105/AJPH.88.11.1603 9807523 PMC1508556

[21] Rowland D Telyukov AV. Soviet health care from two perspectives. Health Affairs (1991) 10:71–86. 10.1377/hlthaff.10.3.71 1748393

[22] Forgotson EH Forgotson J. Innovations and experiments in uses of health manpower: a study of selected programs and problems in the United Kingdom and the Soviet Union. Medical Care (1970) 8:3–14. 10.1097/00005650-197001000-00002 5417721

[23] Mckee M. Understanding population health: lessons from the former Soviet Union. Clin Med. (2005) 5:374–8. 10.7861/clinmedicine.5-4-374 16138493 PMC4954211

[24] GBD 2015 Maternal Mortality Collaborators. Global, regional, and national levels of maternal mortality, 1990–2015: a systematic analysis for the global burden of disease study 2015. Lancet (2016) 388:1775–812. 10.1016/S0140-6736(16)31470-2 27733286 PMC5224694

[25] Global Burden of Disease Child and Adolescent Health Collaboration Kassebaum N Kyu HH Zoeckler L Olsen HE Thomas K. Child and adolescent health from 1990 to 2015: findings from the global burden of diseases, injuries, and risk factors 2015 study. JAMA Pediatr. (2017) 171:573–92. 10.1001/jamapediatrics.2017.0250 28384795 PMC5540012

[26] Atun RA Samyshkin YA Drobniewski F Kuznetsov SI Fedorin IM Coker RJ. Seasonal variation and hospital utilization for tuberculosis in Russia: hospitals as social care institutions. Eur J Public Health (2005) 15:350–4. 10.1093/eurpub/cki018 16030135

[27] Jakovljevic M Vukovic M Fontanesi J. Life expectancy and health expenditure evolution in eastern Europe - DiD and DEA analysis. Expert Rev Pharmacoecon Outcomes Res. (2016) 16:537–46. 10.1586/14737167.2016.1125293 26606654

[28] Tarantola D. A perspective on the history of health and human rights: from the cold war to the gold war. J Public Health Policy (2008) 29:42–53. 10.1057/palgrave.jphp.3200159 18368018

[29] Preker AS Feachem RG Market Mechanisms and the Health Sector in Central and Eastern Europe, Vol. 293 Washington, DC: World Bank Publications (1995). Available online at http://documents.worldbank.org/curated/en/918761468752051137/pdf/multi0page.pdf

[30] Tkatchenko E McKee M Tsouros AD. Public health in Russia: the view from the inside. Health Policy Plan. (2000) 15:164–9. 10.1093/heapol/15.2.164 10837039

[31] Oleszczyk M Švab I Seifert B Krzton-Królewiecka A Windak A. Family medicine in post-communist Europe needs a boost. Exploring the position of family medicine in healthcare systems of Central and Eastern Europe and Russia. BMC Fam Pract. (2012) 13:15. 10.1186/1471-2296-13-15 22409775 PMC3368769

[32] Rechel B McKee M. Health reform in central and eastern Europe and the former Soviet Union. Lancet (2009) 374:1186–95. 10.1016/S0140-6736(09)61334-9 19801097

[33] Sheiman I. New methods of financing and managing health care in the Russian Federation. Health Policy (1995) 32:167–80. 10.1016/0168-8510(95)00734-A 10156637

[34] Sheiman I Shevski V. Evaluation of health care delivery integration: the case of the Russian Federation. Health Policy (2014) 115:128–37. 10.1016/j.healthpol.2013.12.011 24461718

[35] World Health Organization A System of Health Accounts. 2011. 13th Meeting of OECD Health Accounts Experts Paris 4–5 Oct 2011. World Health Organization (2011). Available online at http://www.oecd.org/health/health-systems/48845889.pdf

[36] World Health Organization WHO Global Health Expenditure Database. World Health Organization (2015) Available online at http://apps.who.int/nha/database/Select/Indicators/en

[37] Álvarez-Gálvez J Jaime-Castillo AM. The impact of social expenditure on health inequalities in Europe. Soc Sci Med. (2018) 200:9–18. 10.1016/j.socscimed.2018.01.006 29355829

[38] Eberhardt P Ethnic Groups and Population Changes in Twentieth Century Eastern Europe: History, Data and Analysis. New York, NY: Routledge (2015).

[39] World Health Organization WHO-Health Accounts. (2011). Available online at www.who.int/health-accounts/en/

[40] Schieber GJ Poullier JP Overview of international comparisons of health care expenditures. Health Care Financ Rev. (1989) 1989(Suppl.):1–7. PMC4195146 10313431

[41] Schieber GJ Poullier JP. International health spending: issues and trends. Health Affairs (1991) 10:106–16. 10.1377/hlthaff.10.1.106 1904389

[42] Gerdtham UG Jönsson B International comparisons of health expenditure: theory, data and econometric analysis. Handbook Health Econ. (2000) 1:11–53. 10.1016/S1574-0064(00)80160-2

[43] Getzen TE. Health care is an individual necessity and a national luxury: applying multilevel decision models to the analysis of health care expenditures. J Health Econ. (2000) 19:259–70. 10.1016/S0167-6296(99)00032-6 10947579

[44] Jakovljevic M Arsenijevic J Pavlova M Verhaeghe N Laaser U Groot W. Within the triangle of healthcare legacies: comparing the performance of South-Eastern European health systems. J Med Econ. (2017) 20:483–92. 10.1080/13696998.2016.1277228 28035843

[45] World Health Organization The World Health Report 2000: Health Systems: Improving Performance. World Health Organization (2000).

[46] Jakovljevic M Getzen TE. Growth of global health spending share in low and middle income countries. Front Pharmacol. (2016) 7:21. 10.3389/fphar.2016.00021 26903867 PMC4751681

[47] Murata C Yamada T Chen CC Ojima T Hirai H Kondo K. Barriers to health care among the elderly in Japan. Int J Environ Res Public Health (2010) 7:1330–41. 10.3390/ijerph7041330 20617033 PMC2872331

[48] Jakovljevic M Milovanovic O. Growing burden of non-communicable diseases in the emerging health markets: the case of BRICS. Front Public Health (2015) 3:65. 10.3389/fpubh.2015.00065 25954740 PMC4407477

[49] Ogura S Jakovljevic M Health financing constrained by population aging—an opportunity to learn from Japanese experience. Ser J Exp Clin Res. (2014) 15:175–81. 10.2478/sjecr-2014-0022

[50] Jakovljevic M Yamada T. Editorial: role of health economic data in policy making and reimbursement of new medical technologies. Front Pharmacol. (2017) 8:662. 10.3389/fphar.2017.00662 28983250 PMC5613116

[51] Jakovljevic MB Targeted immunotherapies overtaking emerging oncology market value based growth. JBUON (2015) 20:348–53. 25778340

[52] Jakovljevic M Rankovic A Racic N Jovanovic M Ivanovic M Gajovic O. Radiology services costs and utilization patterns estimates in Southeastern Europe-a retrospective analysis from Serbia. Value Health Regional Issues (2013) 2:218–25. 10.1016/j.vhri.2013.07.002 29702868

[53] Kringos DS Boerma W van der Zee J Groenewegen P. Europe's strong primary care systems are linked to better population health but also to higher health spending. Health Affairs (2013) 32:686–94. 10.1377/hlthaff.2012.1242 23569048

[54] Garber AM Skinner J. Is American health care uniquely inefficient? J Econ Perspect (2008) 22:27–50. 10.1257/jep.22.4.27 19305645 PMC2659297

[55] GBD 2015 SDG Collaborators. Measuring the health-related sustainable development goals in 188 countries: a baseline analysis from the global burden of disease study 2015 Lancet (2016) 388:1813–50. 10.1016/S0140-6736(16)31467-2 27665228 PMC5055583

[56] Jakovljevic MM Ogura S. Health economics at the crossroads of centuries–from the past to the future. Front Public Health (2016) 4:115. 10.3389/fpubh.2016.00115 27376055 PMC4899886

[57] Jakovljevic MM Pejcic A. Growth of global publishing output of health economics in the XXI century: a bibliographic insight. Front Public Health (2017) 5:211. 10.3389/fpubh.2017.00211 28848731 PMC5554506

[58] Field MG The Modern Medical System: The Soviet Variant. Asian Medical Systems: A Comparative Approach. Los Angeles, CA: University of California Press (1976).

[59] Jakovljevic M Varjacic M. Commentary: do health care workforce, population, and service provision significantly contribute to the total health expenditure? An econometric analysis of Serbia. Front Pharmacol. (2017) 8:33. 10.3389/fphar.2017.00033 28220072 PMC5292403

[60] Jakovljevic M Vukovic M Chia-Ching C Antunovic M Dragojevic-Simic V Velickovic-Radovanovic R. Do health reforms impact cost consciousness of Health care professionals? Results from a nation-wide survey in the Balkans. Balkan Med J. (2016) 33:8–17. 10.5152/balkanmedj.2015.15869 26966613 PMC4767315

[61] Tavits M Letki N When left is right: party ideology and policy in post-communist Europe. American Political Sci Rev. (2009) 103:555–69. 10.1017/S0003055409990220

[62] Popovich L Potapchik E Shishkin S Richardson E Vacroux A Mathivet B. Russian Federation. Health system review. Health Syst Transit. (2011) 13:15–217. 22455875

[63] McIntyre D Thiede M Dahlgren G Whitehead M. What are the economic consequences for households of illness and of paying for health care in low-and middle-income country contexts? Social Sci Med. (2006) 62:858–65. 10.1016/j.socscimed.2005.07.001 16099574

[64] Tulchinsky TH Varavikova EA. Addressing the epidemiologic transition in the former Soviet Union: strategies for health system and public health reform in Russia. Am J Public Health (1996) 86:313–20. 10.2105/AJPH.86.3.313 8604754 PMC1380508

[65] GBD 2015 Mortality and Causes of Death Collaborators. Global, regional, and national life expectancy, all-cause mortality, and cause-specific mortality for 249 causes of death, 1980–2015: a systematic analysis for the Global Burden of Disease Study 2015. Lancet (2016) 388:1459–544. 10.1016/S0140-6736(16)31012-1 27733281 PMC5388903

[66] Bonilla-Chacin ME Murrugarra E Temourov M. Health care during transition and health systems reform: evidence from the poorest CIS countries. Soc Policy Admin. (2005) 39:381–408. 10.1111/j.1467-9515.2005.00446.x24843434

[67] Rancic N Jakovljevic M Long term health spending alongside population aging in N-11 emerging nations. East Eur Bus Econ J. (2016) 2:2–26.

[68] Marten R McIntyre D Travassos C Shishkin S Longde W Reddy S. An assessment of progress towards universal health coverage in Brazil, Russia, India, China, and South Africa (BRICS). Lancet (2014) 384:2164–71. 10.1016/S0140-6736(14)60075-1 24793339 PMC7134989

[69] Jakovljevic MB. Resource allocation strategies in Southeastern European health policy. Eur J Health Econ. (2013) 14:153–9. 10.1007/s10198-012-0439-y 23143312

[70] Jakovljevic M Jovanovic M Lazic Z Jakovljevic V Djukic A Velickovic R Antunovic M Current efforts and proposals to reduce healthcare costs in Serbia. Ser J Exp Clin Res. (2011) 12:161–3. 10.5937/sjecr1104161J

[71] McMenamin I Timonen V Poland's health reform: politics, markets and informal payments. J Soc Policy (2002) 31:103–18. 10.1017/S0047279402006517

[72] Sándor J Kosa K Papp M Fürjes G Korösi L Jakovljevic M. Capitation based financing hampers the provision of preventive services in primary health care. Front Public Health (2016) 4:200. 10.3389/fpubh.2016.00200 27679797 PMC5020077

[73] Bryndová L Pavlokova K Roubal T Rokosova M Gaskins M Czech Republic. Health system review. Health Syst Transit. (2009) 11:1–117.

[74] Rao KD Petrosyan V Araujo EC McIntyre D. Progress towards universal health coverage in BRICS: translating economic growth into better health. Bull World Health Organ. (2014) 92:429–35. 10.2471/BLT.13.127951 24940017 PMC4047799

[75] de Marsilllac MelsertII AL BockII AMB The subjective dimension of social inequality: studying life. Educ Res. (2015) 41:774.

[76] Lewis MA Who is Paying for Health Care in Eastern Europe and Central Asia?. Washington, DC: World Bank Publications (2000). Available online at http://siteresources.worldbank.org/ECAEXT/Resources/publications/healthcareinECA2000/Who+Is+Paying+text.pdf

[77] Jakovljevic M Groot W Souliotis K. Health care financing and affordability in the emerging global markets. Front Public Health (2016) 4:2. 10.3389/fpubh.2016.00002 26835444 PMC4720748

[78] Jakovljevic M Lazarevic M Milovanovic O Kanjevac T. The new and old Europe: East-West split in pharmaceutical spending. Front Pharmacol. (2016) 7:18. 10.3389/fphar.2016.00018 26973521 PMC4771948

[79] Global Burden of Disease Health Financing Collaborator Network. Future and potential spending on health 2015-40: development assistance for health, and government, prepaid private, and out-of-pocket health spending in 184 countries. Lancet (2017) 389:2005–30. 10.1016/S0140-6736(17)30873-5 28433260 PMC5440765

[80] Jakovljevic M Potapchik E Popovich L Barik D Getzen TE. Evolving health expenditure landscape of the BRICS nations and projections to 2025. Health Econ. (2017) 26:844–52. 10.1002/hec.3406 27683202

